# Contingent Negative Variation in the Evaluation of Neurocognitive Disorders Due to Possible Alzheimer’s Disease

**DOI:** 10.3390/neurolint16010008

**Published:** 2024-01-11

**Authors:** Arquímedes Montoya-Pedrón, Carmen María Ocaña Montoya, Jorge Esteban Santos Toural, Tania Acosta Lee, Miguel Enrique Sánchez-Hechavarría, Erislandis López-Galán, Gustavo Alejandro Muñoz-Bustos

**Affiliations:** 1 Department of Clinical Neurophysiology, General Hospital “Dr. Juan Bruno Zayas Alfonso”, Santiago de Cuba 90100, Cuba; 2 Hospital Infantil Sur, Santiago de Cuba 90100, Cuba; 3 Telecommunications Department, Universidad de Oriente, Santiago de Cuba 90500, Cuba; jsantos@uo.edu.cu; 4 Departamento de Ciencias Clínicas y Preclínicas, Facultad de Medicina, Universidad Católica de la Santísima Concepción, Concepción 4070129, Chile; 5 Núcleo Científico de Ciencias de la Salud, Facultad de Ciencias de la Salud, Universidad Adventista de Chile, Chillán 8320000, Chile; 6 Laboratorio de Psicología, Departamento de Psicología, Universidad de Concepción, Concepción 4070386, Chile; 7 Facultad de Medicina 2, Universidad de Ciencias Médicas de Santiago de Cuba, Santiago de Cuba 90100, Cuba; erislandislopez@infomed.sld.cu; 8 Escuela de Kinesiología, Facultad de Salud y Ciencias Sociales, Campus El Boldal, Sede Concepción, Universidad de Las Américas, Concepción 4030000, Chile

**Keywords:** Alzheimer’s, neurocognitive disorder, contingent negative variation (CNV)

## Abstract

The usefulness of Contingent Negative Variation (CNV) potential as a biomarker of neurocognitive disorders due to possible Alzheimer’s disease, is based on its possible physiological correlates. However, its application in the diagnostic evaluation of these disorders is still incipient. The aim of this study is to characterize the patterns of cognitive processing of information in the domain of nonspecific global attention, by recording potential CNV in a group of patients with neurocognitive disorders due to possible Alzheimer’s disease. An experimental study of cases and controls was carried out. The sample included 39 patients classified according to DSM-5 with a neurocognitive disorder subtype possibly due Alzheimer’s disease, and a Control Group of 53 subjects with normal cognitive functions. CNV potential was registered using standard protocol. The analysis of variance obtained significant differences in mean values and confidence intervals of total CNV amplitude between the three study groups. The late CNV segment amplitudes makes it possible to discriminate between the level of mild and major dysfunction in the group of patients. The CNV total amplitudes of potential allows for effective discrimination between normal cognitive functioning and neurocognitive disorders due to possible Alzheimer’s disease.

## 1. Introduction

A basic problem in functional brain research is how the multiple serial and parallel neuronal activations necessary to process basic stimuli are integrated and linked. The precise timing and integration of neural activation is vital for effective information processing in the brain [1]. Electrophysiological techniques provide an important number of advantages in the functional exploration of brain activity, highlighting their high temporal resolution and reflecting changes in the dynamic balance of excitation and inhibition processes of brain neural networks, in the range of milliseconds. Consequently, the recording of electroencephalography activity has been widely validated as a direct quantification of neuronal activity and, unlike functional neuroimaging techniques, it is not based on the indirect presumption of the coupling between neural processes and their vascular hemodynamic correlation [2].

Electrical cortical activity may be registered continuously during the baseline resting state. Also, it may be obtained as a time synchronization of neural networks during performance of cognitive tasks or specific sensory stimulation (Event-Related Potentials in cognitive or sensory processes) [3]. Among the main fields of clinical application of event related potential in cognitive processes, its use stands as an endophenotypic biomarker of neurocognitive disorders of an etiological subtype such as Alzheimer’s disease [4].

A growing interest is reported in the application of different types of cognitive Event Related Potentials (ERPs) to the study and classification of neurocognitive disorders possibly of Alzheimer’s etiology, among them P300 Potential, N400 and Contingent Negative Variation (CNV). CNV constitutes a cognitive event-related potential widely used in clinical practice, evaluating unspecific attention and orienting responses. The experimental paradigm that allows generating CNV potential with greater reliability consists of the combination of two stimuli: one of alert (S1) and the other of response (S2); in inter-stimulus interval negative, a deflection of cortical electrical activity is obtained, which constitutes an expression of the level of cortical activation identified as expectance negativity [5].The CNV’s amplitude increases with the occurrence probability of response stimulus, with an increase in discrimination difficulty between the two stimuli (S1, S2) and with a higher motivation level to obtain correct responses.

The potential usefulness of quantifying CNV potential as a biomarker of neurocognitive disorders (NCD) possibly due to Alzheimer’s disease is based on its likely physiological correlates. Brain CNV sources have not been precisely located and an extensive cortical distribution has been described. Experimental animal models have suggested that its generation is associated with activations in pre-striated regions and the prefrontal contralateral cortex to execute movement of the extremities [3]. In humans, experimental evidence suggests that the prefrontal cortex; the bidirectional ipsilaterallong-distance pathways which interconnect uni-polymodal occipito-temporo-parietal cortical areas to prefrontal ones; the premotor, motor, supplementary motor, postcentral and cingulate areas and auditory cortex and its vicinity; and the supplementary sensorimotor area and thalamus have been considered to play a role in the generation of CNV [3,4]. Likewise, it has been reported that CNV results from the activation of a large neural network involving the lateral orbitofrontal, mesial and back areas of the prefrontal cortex, and covers a neural circuit associated with motor preparation [6]. Therefore, CNV reflects the dynamic balance processes between excitation and inhibition that occur in these neural networks, thus making it a valuable tool for investigating cognitive processes. This could be of paramount importance to patients struggling with dementia, as it might improve their diagnosis in the early stages of the disease.

The number of reports evaluating CNV potential in NCD and/or dementia syndromes of the Alzheimer’s type is very small compared to reports found in other types of ERPs that mainly address the P300 potential. This suggests that CNV characterization and its application in cognitive function evaluation is still incipient [6]. As well, contradictory reports have been made regarding the potential value of CNV as a biomarker of NCD due to probable or possible Alzheimer’s disease. Van Deursen et al. [7] report that amplitude of CNV potential does not show significant changes between Alzheimer’s disease patients, those with mild neurocognitive disorder, and healthy control subjects. On the other hand, in the consulted bibliography we found a group of reports that confirm CNV potential value as a marker of NCD due to Alzheimer’s disease. In this regard, Zappoli R. et al. [8] obtained a significant discriminating effect of CNV between healthy and sick subjects with “idiopathic *pre senile initial cognitive decline*”, in what they defined as an initial state of Alzheimer’s disease in correspondence with DSM II1-R and ICD-IO according to updated criteria of that time. These same authors previously published studies that suggest that amplitude of CNV potential is significantly reduced in a group of patients in early clinical or prodromal stages of Alzheimer’s disease. They also reported that reduction in expectancy amplitude negativity reaches greater significance in the late segment of CNV potential [9]. Furthermore, significant correlations between early CNV amplitude and blood flow in the frontal cortex were reported in both vascular dementia and Alzheimer’s disease [10]. The AD patients showed a larger component score for CNV to irrelevant stimuli than the healthy subjects. This suggests that AD patients may have problems in anticipating important stimuli [11]. CNV as part of a combined model predicts which individuals progress to Alzheimer’s disease and which do not [12].

The use of ERPs as markers of different clinical subtypes of neurocognitive disorder is a promising field, but still little explored [13]. Cespon et al. (2015) [14] suggests that these potentials are not useful in the discrimination of clinical subtypes of mild neurocognitive disorder. Nevertheless, Ning N. et al. [15] report a significant decrease in late (_la_CNV) amplitude in people with stuttering disorders and propose it may represent an expression of motor preparation processes for spoken language.

The aim of the present study was to use CNV segmentation to investigate the patterns of cognitive processing of information in the domain of nonspecific global attention and orientation response, in neurocognitive disorders of a possible Alzheimer’s etiology for the two levels of mild and major dysfunction.

## 2. Methods

### 2.1. Place of Study and Participants

This study was carried out in the neurophysiology service of the Juan Bruno Zayas Hospital in Santiago de Cuba, Cuba. Patients were recruited through the dementia clinic at the same hospital between August and October 2022, and healthy subjects were obtained from people who volunteered for the study call. An experimental study of cases and controls was carried out in 39 patients classified according to DSM-5 [16] with NCD due to possible Alzheimer’s disease, and a Control Group of 53 subjects with normal cognitive function.

Group 1. Control: 53 People, 27 male sex, age range between 50–88 years ($\bar{x}=64.72\;\sigma =10.82$), with cognitive function within normal limits.

Group 2. Mild neurocognitive disorder: 15 patients, 8 female sex, age range 53–78 years ($\bar{x}=67.75\;\sigma =8.29$), classified with mild neurocognitive disorder due to possible Alzheimer’s disease according to DSM-5 criteria.

Group 3. Major neurocognitive disorder: 24 patients, 17 female sex, age range between 57–85 years ($\bar{x}=69.61\;\sigma =7.60$), classified with major neurocognitive disorder due to possible Alzheimer’s disease according to DSM-5 criteria.

Participation in the research was carried out under the voluntary principle and the informed written consent of the subjects and/or their legal representatives. Prior to its completion, research was approved by the Ethics Committee of the Juan Bruno Zayas Clinical Surgical Hospital of Santiago de Cuba, (protocol code No. 24-2016 and date of approval 24 November 2016).

Exclusion criteria for all groups included clinical (or imaging) evidence of stroke, head trauma, Parkinson’s disease, or any other neurological or psychiatric disorders; HIV/AIDS; and reversible dementias, as well as treatment with benzodiazepines, antipsychotic, or antiepileptic medications. Additional exclusion criteria were severe cardiovascular disease, a history of substance abuse, and/or other serious system diseases (e.g., malignancy, uncontrolled hypertension, neuropathy or seizure disorders).

The sample size was selected in such a way that it complied with the recommendations of Peacock et al. [17] for comparisons of means. Taking a standard deviation of 7 microvolts in CNV amplitude, a minimum mean difference of 6 microvolts, and a test power of 90%, the number of subjects required is 58. This value was exceeded. The ratio between cases and controls also complies with the recommendations of Peacock et al. [17], where it is stated that there should not be more than three controls per case. It was also determined that both cases and controls must be matched in relation to risk factors. In this research, age matching was prioritized since this was the main risk factor.

### 2.2. Neuropsychological, Neurological and Clinical Assessment

Neuropsychological assessment included multiple cognitive domains (complex attention, memory and learning, executive functions, language, motor and perceptual functions). For an in-depth analysis of executive functions, specific neuropsychological tests were used, including the Stroop test [18], Trail Making Test Part B [19], Clock Drawing Test [20], FAS Verbal Fluency Test [21], animal categorization test [22], reverse Digit Span Test [23], Wisconsin Test [24], direct Digit Repetition Test [25], and the 10-Word List Learning Test [26]. Motor-perceptual abilities, praxis, and gnosis were assessed using a neurological clinical examination. In all subjects, the neuropsychological assessment was integrated with the neurological clinical evaluation, which also encompassed interviews with relatives or caregivers and the patient’s response to challenging scenarios. To define the etiological diagnosis, a neurological and neuropsychiatric clinical evaluation was performed. In addition, neuroimaging studies (Skull Nuclear Magnetic Resonance or Computerized Axial Tomography) and a quantitative electroencephalogram were recorded. Patients with vascular lesions, cerebral electrical patterns compatible with focal cortical dysfunctions and/or clinical signs of focal neurological lesions or neuropsychiatric disorders were excluded.

### 2.3. Electrophysiological Assessment

Contingent Negative Variation: registered according to standardized protocols. Stimulation paradigm auditory alerts stimulus (S1): 3 kHz tone at 80 (dB) decibels “sound *pressure level*” (spl). Visual response stimulus (S2): (LED: Light-Emitting Diodes), frequency: 10 Hz, binocular inter-stimulus interval of 2 s. Manual response: recorded at frontal and central midline EEG derivations (Fz (+)−Cz (+)), references in both mastoids (A1 + A2). Time analysis: 5 s. Bandwidth: 0.03–20 Hz. Sampling frequencies: 200 Hz. Sensitivity 50 µv/division, 20 averages.

The CNV segmentation was carried out in the inter stimulus interval: early CNV (eCNV), between 500–1000 ms after S1; central CNV (cCNV), between 1000–1500 ms; and _la_CNV between 1500–2000 ms before S2 response stimulus. Maximum amplitude in each segment and total maximum amplitude are measured, in respect to 100 ms prior to S1 baseline.

### 2.4. Statistical Analysis

Statistical analysis was performed using JASP software (version 0.16; https://jasp-stats.org (accessed on 31 January 2023)). A *p*-value less than 0.05 was considered statistically significant. Descriptive statistics were reported as means, standard deviations (SD), inferior limits, superior limits, observation number, and percent as appropriate. A univariate analysis was performed with the Chi-squared (χ^2^) test to establish the association between the neuropsychological profile and neurocognitive disorder of the patients group, and the ANOVA model was applied to evaluate cognitive functioning level effect for tCNV amplitude and each CNV’s potential segment. A multivariate analysis was performed using MANOVA to measure the effect of the level of cognitive functioning in CNV amplitude on its three segments, and evaluated in a comparative way only within the neurocognitive disorder patient groups due to possible Alzheimer disease (mild and major). Octave software 2019 (version 5.1.0; https://www.gnu.org/software/octave/download.html (accessed on 15 January 2023)) was used to prepare the graphs of CNV potentials’ grand averages.

## 3. Results

Table 1 illustrates the neuropsychological profile of patients with NCD, possibly the Alzheimer’s disease subtype. Results show that the main characteristic of a neuropsychological profile of mild NCD possibly due to Alzheimer’s disease is related to executive function (with a decrease in 93.33% of cases). The complex attention domain constitutes the second most affected domain, and the third most affected domain was language (26.66%). An interesting aspect was the high percentage of normality in the memory and learning domain (93.33%). The neuropsychological profile of perceptual and motor functions is also characterized by a high prevalence of normal functioning.

In the group of patients with major NCD due to possible Alzheimer’s disease, the level of deficient functioning predominates in all explored domains except for the perception domain and motor functions, where normal functioning predominates in 58.33% of cases and decreased in 33.33% of cases. It is noted that a deficient functioning level in executive functions is the one that occurs most frequently in this patient group.

Table 2 shows the results of the evaluation of cognitive functioning level effect for tCNV amplitude and each CNV’s potential segment. The ANOVA analysis of variance demonstrated that cognitive level has a statistically significant effect on tCNV amplitude (ANOVA *p* = 0.000). Likewise, a significant effect of cognitive level is obtained on amplitude for each one of the CNV potential segments (ANOVA *p* = 0.000).

**Figure 1:**
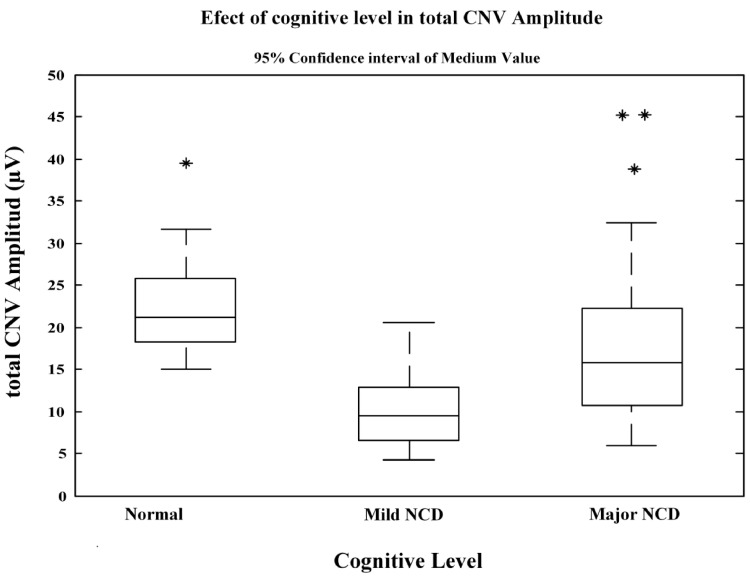
Effect of cognitive level in total CNV amplitude. All asterisk symbol (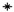) indicates outliers. The top and bottom lines are the whiskers and represent the maximum and minimum values determined by multiplying the value of the interquartile range by 1.5.

In both patient groups affected with neurocognitive disorders, mean values of tCNV amplitude were significantly reduced with respect to the values of the healthy control group. Reduction in CNV amplitude reached its greatest magnitude in the mild NCD group ($\bar{x}=10.63\;\mu V\;\sigma =4.88\;\mu V$). Meanwhile, in the major NCD group, a moderate reduction in tCNV amplitude was recorded ($\bar{x}=17.93\;\mu V\;\sigma =9.45\;\mu V$). The mean values and confidence intervals of tCNV amplitude are very different between the three study groups and the overlap degree between confidence intervals limits is very small. This result shows that tCNV amplitude has a statistical distribution that efficiently discriminates between normal cognitive functioning and mild or major neurocognitive disorder due to a possible Alzheimer’s disease subtype (Figure 1).

**Figure 2:**
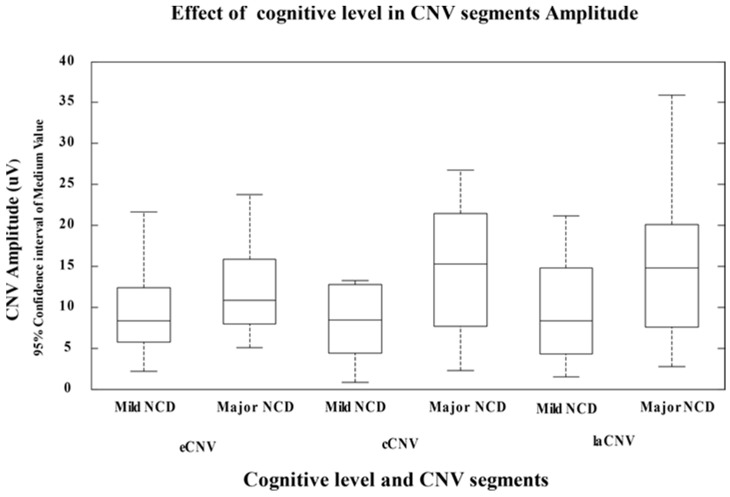
Effect of cognitive level in total CNV segments amplitude in both groups of patients with NCD due to possible Alzheimer’s disease (eCNV: early CNV, cCNV: central CNV, laCNV: late CNV).

Figure 2 shows the analysis of variance (MANOVA) results of cognitive functioning level effect in CNV amplitude on its three segments, and evaluated in a comparative way only within the neurocognitive disorder patient groups due to possible Alzheimer disease (mild and major). Note that there are differences in CNV amplitude between both cognitive dysfunction levels that reach statistical significance only in _la_CNV amplitude late segment (*p* = 0.035). On the other hand, there are no significant differences in CNV amplitude in early (*p* = 0.196) and central (*p* = 0.056) segments for each level of cognitive dysfunction.

**Figure 3:**
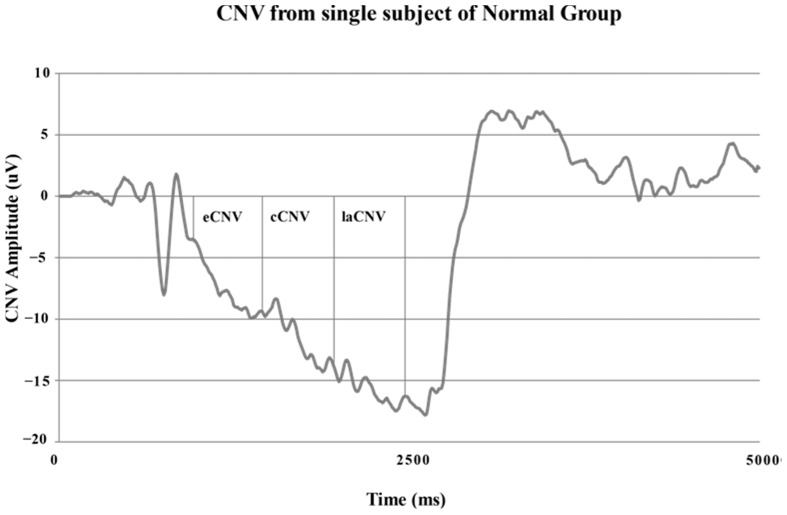
Single record obtained from subjects of the normal control group (eCNV: early CNV, cCNV: central CNV, laCNV: late CNV).

Figure 3 shows a single CNV potential recorded from a healthy normal subject. This is the typical electrophysiological pattern recorded in normal control group subjects. The registered clear definition of expectancy negativity and orientation (O) and responses (R) waves can be observed. The evaluated segments in the CNV potential are also illustrated.

**Figure 4:**
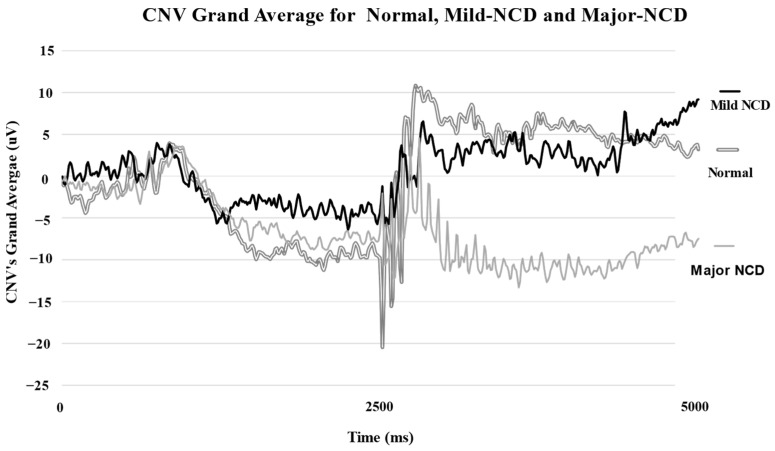
CNV potential grand averages for normal, mild and major NCD groups.

Figure 4 illustrates the grand average of all signals recorded for each study group. It confirms the statistical results regarding the mean value of total CNV amplitude and demonstrates the reported changes obtained in CNV segments. It highlights the negative deflection of the postimperative segment in the major NCD group. In contrast, normal and mild NCD groups obtained a positive baseline deflection in the postimperative segment.

## 4. Discussion

### 4.1. Neuropsychological Profile

The results show that the main characteristic of a neuropsychological profile of mild NCD due to a possible Alzheimer’s disease subtype is the involvement of the executive functions. Major NCD is characterized by poor functioning in all domains, particularly in executive functions. Likewise, perceptual and motor functions obtain a functioning level that predominates between normal and decreased.

The consulted literature confirms that for the diagnosis of neurocognitive disorders, memory, learning and language domains have been extensively studied [19]. However, other authors have reported that involvement of these domains tends to be delayed, and neuropsychological examination can demonstrate early-stage disorders predominantly in the executive functions sphere [27,28].

In correspondence with clinical subtypes of the mild cognitive impairment classification proposed by Petersen et al. [28] (amnesic, multi domain amnesic, non-amnesic, non-amnesic multi domain), the neuropsychological profile that characterizes the patient groups studied corresponds with the non-amnesic multi domain type.

### 4.2. Utility of CNV Potential in Neurocognitive Disorders Due to Possible Alzheimer’s Disease

Addressing the interpretation of CNV findings, our results showed that the amplitude of expectancy negativity is significantly reduced in patients belonging to the neurocognitive disorder groups due to possible Alzheimer’s disease in comparison to the healthy control subjects.

As previously mentioned, there is no consensus in the consulted bibliography on the possible usefulness of the CNV potential in neurocognitive disorder diagnosis (see introduction). However, the current trend leans in favor of its usefulness in evaluation of these disorders, which is consistent with our results. In more recent studies, Chapmam et al. 2018 [29] reported that CNV reaches an optimal discrimination power as a biomarker of Alzheimer’s disease, applying principal component analysis, integrating spatial distribution and temporal activation patterns. The authors report ROC curves with excellent performance that confirm the discriminative power of the CNV potential in diagnosing Alzheimer’s disease, providing a posterior probability model that allows defining the probability of proximity of an individual being classified as a sick or healthy control.

### 4.3. CNV Potential and Underlying Neural Processes

In our results an interesting finding that should be analyzed is the fact that the CNV amplitude reaches its lowest value in the mild neurocognitive disorder patient group, while expectancy negativity obtains mean amplitude values of greater magnitude in the major neurocognitive disorder group.

In this regard, there are some reports that describe a similar effect in expectancy negativity and readiness potential amplitudes, and attribute it to a possible compensatory effect with greater severity of cognitive impairment. It implies the recruitment of a greater number of cortical neurogenerators associated with expectancy negativity [13]. Some individuals with AD have been reported to have greater cognitive efficiency than others despite a relatively similar level of functional impairment, and the way the brain overcomes damage may explain how these individuals maintain good cognitive performance. Functionally, AD can be expected to reduce neuronal activity in damaged brain regions. Increased neural activity in other brain regions could allow some individuals with AD to compensate and thus perform perceptual-cognitive tasks with an accuracy closer to that of normal, healthy elderly control subjects [30]. Moreover, this possible compensatory effect has also been described for other cognitive event related potential types, mainly the P300 potential [31].

Another possibility to explain the greater amplitude of the CNV, the post-imperative negativity, and greater cognitive impairment present in patients with AD, could be an alteration in the circuits that modulate cortical activity. The most extensive of these circuits is the cholinergic component of extrathalamic cortical projections, which has been implicated in the modulation of a set of behavioral states including attention, arousal, memory, learning and sleep. The nucleus basalis-Ch4 complex stands out as one of the structures of the cholinergic component of extrathalamic cortical projections that presents a high degree of vulnerability to tauopathy and neurofibrillary degeneration, which implies a significant functional impact on cognitive function in Alzheimer’s pathology. Circumstantial evidence for the relevance of cholinergic denervation to the cognitive and behavioral changes in AD comes from the symptomatic improvements that have been achieved through the use of cholinomimetic drugs [32]. However, there is some evidence that anticholinergic medications commonly used to treat early-stage AD have little influence on ERPs [33]. Furthermore, the exact contribution of cholinergic denervation to cognitive impairment is almost impossible to determine, because it unfolds on a background of amyloid deposition and neurofibrillary pathology elsewhere in the brain [32].

The amplitude increase of expectancy negativity in CNV potential has also been associated with normal aging processes. In a study that evaluates differential age effect and executive functions in CNV resolution, Dirnberger G. et al. [34] report that, contrary to what was expected, CNV amplitude increases with normal aging. This author formulates the hypothesis that a decrease in executive functions and a focus on inhibition processes, causes a conditioned need for greater executive control in the CNV’s experimental paradigm execution. As a result, the cortical activation process related to CNV generation could be associated with increased neurogenerator recruitment. Carsson M. et al. [35] also report a similar effect of physiological aging on CNV amplitude.

Other studies have obtained a contradictory result to those previously mentioned in respect to cognitive effect decline associated with physiological aging in CNV amplitude. Ya-Nan Niu et al. [36] showed that in a group of people with advanced age (mean 69 years), amplitude of expectancy negativity is significantly reduced when it is associated with decreased motor functions and decreased performance in action and reaction mechanisms.

In the present study, results from a particular context concerning cognitive level effect in CNV amplitude, age influence should not be considered as statistically significant because the three groups included matched for this variable, with medium values and very similar confidence intervals for each group. As well, there were no significant impairments in the motor and perceptual functions domains. Therefore, compensatory effect theory with a greater neurogenerator recruitment for cognitive deficit as a result of greater severity seems to be the hypothesis that best explains our results. Further studies combining functional neuroimaging techniques and topographic distribution maps with inverse solution models are required to confirm or reject this hypothesis.

### 4.4. Electrophysiological Patterns Recorded in CNV Potential

It is important to emphasize that our results suggest that regardless of their possible functional correlations, statistical distribution of tCNV amplitude potential and each of its segments, allows us to precisely discriminate between normal cognitive functioning and mild or major neurocognitive disorders due to possible Alzheimer’s disease. Also of note is that their discriminatory power is more efficient for distinguishing between healthy controls and mild neurocognitive disorder, which increases their likelihood of being used as a complementary diagnosis biomarker in milder and early stages of Alzheimer’s disease.

Finally, we refer to possible pathophysiological correlates of the electrophysiological patterns recorded in CNV potential in neurocognitive disorders that are possibly due to Alzheimer’s disease. In correspondence with the functional significance that is consistently attributed to CNV potential generators in the consulted bibliography [37], our results suggest that in subjects with neurocognitive disorder of a possible Alzheimer’s disease subtype, there is consistent electrophysiological evidence that shows a decrease in nonspecific attention processes and orientation response, a reduction in intensity and maintenance of the cortical activation level associated with expectancy processes, and signs of dysfunction in the anticipation or preparation processes for a motor response.

The electrophysiological pattern registered in CNV potential is compatible with the clinical neuropsychological profile of executive function deficits that we report in this study sample, considering that the experimental task in which this potential is obtained directly explores attention to executive processes and orientation response. This hypothesis is supported by a study carried out by Daniela Mannarelli [38], where she examined the right prefrontal dorsolateral cortex’s role during attention processing of stimuli in a double-choice reaction time experiment, recording CNV before and after causing transient right dorsolateral prefrontal cortex inhibition by repetitive transcranial magnetic stimulation (rTMS) at a 1 Hz frequency. A CNV response attenuation or suppression was obtained in reaction to rTMS, which demonstrates the participation of this cortical area in ERP CNV generation.

### 4.5. Limitations and Future Directions

Our study has some limitations, mainly associated with the relatively low number of patients. In addition, there was a considerable amount of variability in the methodology to obtain the CNV and in its processing, especially in segmentation. Further studies combining functional neuroimaging techniques and topographic distribution maps with inverse solution models are required to determine patterns by brain regions.

## 5. Conclusions

In the light of the results of the present study, we conclude that tCNV potential amplitude allows the efficient discrimination between normal cognitive functioning and neurocognitive disorder due to possible Alzheimer’s disease. Evaluation of CNV amplitudes by segments also allows the establishment of the cognitive impairment level (mild or major) and inferences about processes of non-specific global attention functional status, orienting response mechanisms and preparation motor response. Furthermore, CNV potential could reflect the brain plasticity, cognitive compensation or alteration in the circuits that modulate cortical activity. Therefore, quantified parameters in CNV potential can be used as classifying electrophysiological biomarkers of presence and level of cognitive dysfunction possibly due to an Alzheimer’s disease etiology.

## Figures and Tables

**Table 1:** Neuropsychological profile of patient groups affected by neurocognitive disorder due to possible Alzheimer’s disease.

	Cognitive Level	Complex Attention		Executive Functions		Learning and Memory		Language		Motor Functions and Perception	
		*n*	%	*n*	%	*n*	%	*n*	%	*n*	%
Mild NCD	Normal	9	60	1	6.66	14	93.33	11	73.33	14	93.33
	Decreased	6	40	14	**93.33**	1	6.66	4	26.66	1	6.66
	Total	15	100	15	100	15	100	15	100	15	100
Major NCD	Normal	1	4.16	-	-	-	-	3	12.5	14	58.33
	Decreased	7	29.16	1	4.16	11	45.83	8	33.33	8	33.33
	Deficits	16	66.66	23	**95.83**	13	54.16	13	54.16	2	8.33
	Total	24	100	24	100	24	100	24	100	24	100
		*p* = 0.000		*p* = 0.000		*p* = 0.000		*p* = 0.000		*p* = 0.067	

*n*: Observation number; NCD: Neurocognitive Disorder.

**Table 2:** Effect of cognitive level in Contingent Negative Variation amplitude.

Amplitude	Cognitive Level	*n*	Mean (µv)	SD	IL	SL	ANOVA Sig.
Total tCNV	Normal	50	23.79	6.52	21.94	25.64	*p* = 0.000
	Mild	10	10.63	4.88	7.13	14.13	
	Major	20	17.93	9.45	13.50	22.36	
Early eCNV Segment	Normal	50	22.42	5.77	20.34	24.93	*p* = 0.000
	Mild	10	9.50	5.64	5.45	13.54	
	Major	20	13.36	8.27	9.48	17.23	
Central cCNV Segment	Normal	50	24.57	7.23	22.05	26.17	*p* = 0.000
	Mild	10	8.13	4.22	5.11	11.15	
	Major	20	15.01	10.41	10.13	19.88	
Late _la_CNV Segment	Normal	50	23.98	6.69	22.15	25.74	*p* = 0.000
	Mild	10	8.68	5.67	4.62	12.73	
	Major	20	15.44	8.72	11.35	19.52	

*n*: Number, SD: Standard deviation, IL: Inferior limits, SL: Superior limits, Sig.: Statistics significance.

## Data Availability

Data is contained within the article.
